# Extracts from a Lecture “On the Present Position in Europe of Some of the Most Interesting and Important Points of Modern Surgery,” Recently Delivered as an Introductory Discourse

**Published:** 1852-01

**Authors:** Thomas D. Mütter

**Affiliations:** Professor of Surgery in Jefferson Medical College, Philadelphia


*Extracts from a Lecture "On the present position in Europe of some of the most interesting and important points of Modern Surgery," recently delivered as an Introductory Discourse.* By THOMAS D. MÜTTER, M. D., Professor of Surgery in Jefferson Medical College, Philadelphia.

*Anesthesia.*—My attention, naturally enough, was first directed to the subject of anesthesia. I need not, on this occasion, enter upon the history of this purely *American* discovery. I repeat purely *American discovery*, for, notwithstanding the attempts made by some, to give the credit of this most valuable of all modern improvements in surgery to Europeans, we have yet positive evidence of its being in truth an offspring of the New World. I do not pretend that efforts to mitigate the suffering attendant upon surgical operations were not made long before the introduction of the new process by the inhalation of some sedative agent. In fact, in all ages, surgeons have endeavored to attain, by various measures, this truly important end. Thus we read in Middleton's tragedy of "Woman, beware Woman," published in 1657,

> "I'll imitate the pities of old Surgeons
> To the lost limb; who, ere they show their art,
> Cast one asleep; then, cut the diseased part."

But I contend, that we have no authority for supposing that prior to the introduction of ethereal inhalations in this country, any agent of the same class had ever been elsewhere practically employed.

It is true that Beddoes and Davy had suggested and even used the nitrous oxide gas, with the view of producing anesthesia, but the experiment attracted little notice and was soon forgotten; and to the American really belongs the honor of having brought to light the immense value of anesthetic agents, in the treatment of painful disorders, or the performance of surgical operations.

Having said this much in defence of our rights, I may proceed to state in what estimation the measure is held abroad, and a few words will suffice to do this.

In England, Scotland and Ireland, and on every portion of the continent of Europe, it would appear, that no surgeon of any grade, high or low, pretends to practice his profession without the constant use of some anesthetic agent. When I asked my distinguished friends in London and Paris, if they employed the measure with the same degree of confidence as at first, they seemed surprised at the question, and unhesitatingly declared, that no surgeon would presume to perform a serious operation without first bringing his patient into a state of anesthesia, provided always, there was nothing present to contraindicate the production of this condition. While there exists some difference of opinion as to the best agent to be used, there is none upon the great point of the value of the measure in the practice of surgery. The agent usually employed is *chloroform*, a fluid first used to any extent by Dr. Simpson, of Edinburgh; and it would appear that very few cases of serious consequences resulting from its employment, have been met with either in public or private practice. With us, however, the reports are not so satisfactory, and hence, some, myself among the number, still prefer the original preparation of sulphuric ether, or else a mixture of the two forming a new fluid called *chloric ether*. Attempts have been made to substitute other fluids, but up to the present time *ether* and *chloroform* are considered by far the safest and most to be relied upon.

The *Bromohydric Ether*, discovered by M. Robin of Paris, has recently attracted some attention, but is not at all in general use.

But, general anesthesia is not alone resorted to. An attempt to obtain a similar condition *locally*, is also a favorite measure. Up to the present moment, however, the experiments with this view, have not been as satisfactory as could be desired. The famous "*Eau Hollandaise*," or "*Ether Chlorohydrique Chloré*," from which so much was anticipated, seems to have proven a total failure, while the "*frigorific mixture*" of Mr. Arnott has likewise been "found wanting" in many of the cases to which it has been applied.

I cannot attempt in this place to indicate the method of using anesthetic agents, nor can I point out the cases most proper for their application. These matters must be left to the general lectures and to the clinic, in which an ample field for observing the practical working of the measure will be afforded you.

Before leaving the subject, I may remark, that in *France* at least, the use of chloroform or ether is chiefly confined to surgical practice, very few using anesthetic influence in obstetrics. In Great Britain, on the other hand, it is much employed in both departments of the profession.

*The Microscope.*—The next point of interest to which my attention was directed, was the influence of microscopic observations in surgery. In *Anatomy* and *Physiology* so much has been gained by the use of the microscope, that I was led to hope that our department had also been enriched through the agency of this most wonderful instrument—nor was I disappointed. In the study of *malignant growths*, much has been discovered both novel and practically useful. Again, in the examination of *secretions*, the microscope has proved of great value. For example, we are able to decide in cases of deep ulcer over a bone, by examining the pus, whether or not the bone is diseased. The presence of pus in the sputa can only be detected by the microscope. It is also of great value in the diagnosis of certain diseases of the *kidney and bladder*. Without it, Mr. Quekett could never have made the wonderful discovery that a *urinary calculus*, a solid stone, is in *reality organized*; and by its use alone can we ever arrive at a correct classification of *Tumors*. The application of the microscope in surgical pathology, however, is still almost an untrodden field, and he who now determines to enter upon its cultivation, will most assuredly reap a rich harvest of renown for himself, and confer lasting benefits upon humanity and science.

*Wounds.*—The next subject of interest, to which I shall direct your attention, is the manner in which extensive wounds are dressed, at the present time, in Europe, and you will naturally enough be surprised to learn that in a matter of such vital importance, there should exist any diversity of opinion among surgeons as to the proper method of treatment; and yet there is scarcely a point in practical surgery, that has elicited more controversy and discussion. The *French*, with but very few exceptions, still adhere to the original views of some of their older authorities, and unite all extensive wounds by the *second intention* of Hunter; while the British surgeons, like most of us, adopt a plan directly the reverse, and endeavor to obtain, as far as possible, union by the

*first intention* of Hunter, or simple adhesion. It afforded me no slight gratification to find, that the principles I have so often inculcated here, in reference to this subject, should be those upon which the practice of such men as Brodie, Lawrence, Stanley, Guthrie, Travers, Furgusson, Phillips, and others of high reputation, have for many years been based, and I was thus fully convinced of the propriety of attempting, when the case justifies such an attempt, the immediate union of a wound. I cannot, at this time, present you with the arguments advanced by the French for adhering to the reverse of this treatment, but on a proper occasion they will all be fully explained.

From what I could learn, the continental surgeons, out of France, are gradually adopting the modern English and American method; and instead of covering up their wounds with great bundles of charpie, or that filthy abomination, a poultice, apply the lightest dressing, and frequently employ nothing but *cold water*, as recommended especially by many of the older surgeons, and more recently by Larrey, McCartney and Liston.

*Traumatic Hemorrhage.*—In the management of *traumatic hemorrhage*, there has been little or no improvement. In fact, surgeons have long since settled down upon the great indications to be observed in such cases, and hence there is but little room for improvement. *Forced flexion* in wounds of the extremities, however, deserves notice and may in many cases prove eminently serviceable. This new measure appears first in the "Anatomie Chirurgicale" of Malgaigne, and has been successful in some few cases, especially in one reported by M. Durwell in the London Medical Gazette.

*Syphilis.*—While in Paris my attention was especially directed to the subject of syphilitic diseases, from the circumstance that the highest authority upon this class of affections still exercises his functions as professor in the "Hospital du Midi." Ricord, by birth a Baltimorean, but for many years a resident of Paris, has long since rendered himself famous in the management of the class of diseases just referred to, and I found that his views, promulgated, some of them, many years since, have undergone very little modification. He still uses mercury in primary syphilis, and considers it the only true and safe remedy. The preparation employed is the proto-ioduret, and my own observations agree with his, in establishing this as the best of all the mercurials, in the treatment of indurated chancre or bubo. *Mercurial fumigation* he also recommends in the strongest terms, as a safe and speedy method of bringing a patient under the influence of the specific.

In phagedenic chancre, *iron* is beyond all question the remedy to be employed internally, while mercury here is almost sure to act as a poison.

Tertiary syphilis he still treats with iodide of potassium as his sheet anchor, and contends, that to be of permanent benefit, it must be administered in large doses.

*Cancer*, in all its phases, has recently been closely investigated by Muller, Langenbeck, Carmichael, Bennett, Quekett, Walshe, Simon, Rokitansky, Libert, and others, but I fear much remains to be done ere we arrive at its true origin and proper treatment. No question seems to exist as to our power of communicating the disease by inoculation. If, for example, we mix a little cancerous matter with water, and inject the mixture into the venous circulation of any animal, we to a certainty induce cancerous deposits in different parts of the body, and especially in the pulmonary veins. The truth of this statement has recently been verified by my friend Dr. Leidy, of this city, who has succeeded in establishing the disease by inoculation even in cold blooded animals; for instance, a frog. With respect to some of the various attempts recently made to cure the disease radically, the plans of Jobert, Lisfranc, Dieffenbach, Phillips, and Arnott, appear to have attracted most attention.

The method of Jobert, which consists in the application of a ligature to all the principal arteries supplying the tumor, and the division of its nervous filaments, seems to have acquired no great reputation, and I scarcely heard it alluded to by the surgeons of London and Paris. The same may be said of the process of Lisfranc, which proposes in cases of superficial cancer in any organ, the removal of the *diseased tissue*, either with the ligature or knife, leaving the organs upon which it happens to be located untouched. The method of Dieffenbach, Phillips, or Martinet de la Cruse, for all claim the merit of the invention, differs from the ordinary operation in this: instead of allowing the wound made during the removal of the tumor to heal by granulation, which is usually permitted to a certain extent in all cases of extensive dissection, a flap of sound skin is taken from the adjacent parts and brought over the raw surface, so that union takes place, and thus prevents the granulating process. It is supposed by the authors of this plan, that the application of the healthy skin to the surface from which the cancerous mass has been removed, will so change the vital action in the part, that health will take the place of disease, and hence a return of the complaint be effectually prevented. But unfortunately, experience is against the operation, and if cancer is a constitutional affection, as it often is, it is difficult to imagine that it could prove so useful as we have been led to suppose. I have, myself, tried the experiment in several cases, but the disease has invariably returned, and I find such to have been the result in the practice of others. The operation will, in all probability, be speedily forgotten, along with a host of other "novelties" that are fast wending their way to the "tomb of all the Capulets!" Mr. Arnott, of London, has recently revived the method of *Recamier*, which consists in the methodic and continued application of pressure to the diseased mass. Experience, so far at least, is against this measure, but in hopeless cases, those, for instance, in which the knife promises nothing, it may be employed, as it will serve to satisfy the patient in part, and prevent, to a certain degree, that terrible sickness of heart that overwhelms a poor sufferer when utterly abandoned by the surgeon.

Recently, Dr. James Arnott, of Brighton, has published some interesting facts which go to prove the value, as a *palliative measure* at least, of the application to cancerous tumors, of a very low degree of temperature, the parts being in fact *frozen*, or nearly so. If his statements are to be relied upon, this is certainly a measure deserving the notice of surgeons.

*Pleurisy.*—An operation altogether novel in the disease for which it was practised, has been introduced by Professor Trousseau of Paris, one of the most distinguished practitioners of that city of of eminent medical men. Professor Trousseau told me that he has succeeded in relieving several patients who were almost in articulo mortis; and I have myself known it to accomplish the same end. The operation is nothing more than the evacuation of the fluid in cases of *acute pleurisy*. You are aware that the secretion is here often exceedingly rapid, and unless the lung be relieved, the patient must die of suffocation. When, therefore, you find a patient thus situated, recollect that paracentesis thoracis, promptly performed, will probably afford immediate relief.

*Injection of Joints.*—Some of you are doubtless aware of the tedious nature of certain chronic inflammatory affections of the joints, especially of the larger ones. Now it has been proposed and the experiment repeatedly tried, to *inject* the *cavity* of the joint diseased, just as we would the tunica vaginalis in hydrocele, with some stimulating fluid, with the view of causing a new action in the secreting surface, by which either adhesion would be accomplished, or a check put to the excessive secretion of the fluid. In England I found the surgeons generally opposed to the operation, as one fraught with great danger, but in Paris it seemed to be quite a popular measure. Velpeau and Jobert both advise it, but I must confess that I saw nothing to induce me to join in this opinion. It is beyond question a most serious operation, and has caused the death of more than one unfortunate patient. The fluid used by the French surgeons is the *tincture of iodine*, and certainly this is much the least hazardous agent.

*Ascites.*—Attempts to cure *ascites* on the same principle, have recently been made by Drs. Dieulafoy, Leriche of Lyons, and Bonnet. *Five* cases of *cure* resulting from iodine *injections*, are reported by the first named gentleman; and the last gives a history of *fifteen*, treated by the injection of *gases*, *water*, *iodine*, and other fluids, *fourteen* of which recovered, only one dying. This is certainly most astonishing success, but the practice of others has proved by no means so devoid of risk. In fact just before I reached Paris, a patient upon whom the operation had been performed, perished most miserably.

*Excision of the Joints.*—In diseases of the joints, complicated with serious lesion of the bones, the operation of excision is becoming quite a favorite measure, particularly in England. The head of the femur, the elbow joint, and even the knee, had been removed just before I reached London, and I was told that success followed each operation. This measure is not, however, a novelty, as it was recommended long since by White and Moreau, and has from time to time been performed by surgeons both at home and abroad.

(To be continued.)

